# Selective Targeting of Class I HDAC Reduces Microglial Inflammation in the Entorhinal Cortex of Young APP/PS1 Mice

**DOI:** 10.3390/ijms24054805

**Published:** 2023-03-02

**Authors:** Chunyang Wang, Di Shen, Yingqiu Hu, Jie Chen, Jingyun Liu, Yufei Huang, Xuebin Yu, Haiying Chu, Chenghong Zhang, Liangwei Yin, Yi Liu, Haiying Ma

**Affiliations:** 1 Department of Histology and Embryology, College of Basic Medical Sciences, Dalian Medical University, Dalian 116044, China; 2 Department of Oncology, Dalian Municipal Central Hospital, Dalian 116089, China; 3 Department of Neurology, Dalian Municipal Central Hospital, Dalian 116089, China

**Keywords:** Alzheimer’s disease, β-amyloid, HDAC inhibitor, synaptic protein, entorhinal cortex, inflammation

## Abstract

BG45 is a class Ⅰ histone deacetylase inhibitor (HDACI) with selectivity for HDAC3. Our previous study demonstrated that BG45 can upregulate the expression of synaptic proteins and reduce the loss of neurons in the hippocampus of APPswe/PS1dE9 (APP/PS1) transgenic mice (Tg). The entorhinal cortex is a pivotal region that, along with the hippocampus, plays a critical role in memory in the Alzheimer’s disease (AD) pathology process. In this study, we focused on the inflammatory changes in the entorhinal cortex of APP/PS1 mice and further explored the therapeutic effects of BG45 on the pathologies. The APP/PS1 mice were randomly divided into the transgenic group without BG45 (Tg group) and the BG45-treated groups. The BG45-treated groups were treated with BG45 at 2 months (2 m group), at 6 months (6 m group), or twice at 2 and 6 months (2 and 6 m group). The wild-type mice group (Wt group) served as the control. All mice were killed within 24 h after the last injection at 6 months. The results showed that amyloid-β (Aβ) deposition and IBA1-positive microglia and GFAP-positive astrocytes in the entorhinal cortex of the APP/PS1 mice progressively increased over time from 3 to 8 months of age. When the APP/PS1 mice were treated with BG45, the level of H3K9K14/H3 acetylation was improved and the expression of histonedeacetylase1, histonedeacetylase2, and histonedeacetylase3 was inhibited, especially in the 2 and 6 m group. BG45 alleviated Aβ deposition and reduced the phosphorylation level of tau protein. The number of IBA1-positive microglia and GFAP-positive astrocytes decreased with BG45 treatment, and the effect was more significant in the 2 and 6 m group. Meanwhile, the expression of synaptic proteins synaptophysin, postsynaptic density protein 95, and spinophilin was upregulated and the degeneration of neurons was alleviated. Moreover, BG45 reduced the gene expression of inflammatory cytokines interleukin-1β and tumor necrosis factor-α. Closely related to the CREB/BDNF/NF-kB pathway, the expression of p-CREB/CREB, BDNF, and TrkB was increased in all BG45 administered groups compared with the Tg group. However, the levels of p-NF-kB/NF-kB in the BG45 treatment groups were reduced. Therefore, we deduced that BG45 is a potential drug for AD by alleviating inflammation and regulating the CREB/BDNF/NF-kB pathway, and the early, repeated administration of BG45 can play a more effective role.

## 1. Introduction

Alzheimer’s disease (AD) is a progressive neurodegenerative disorder with declining cognitive function associated with age [1]. Morbidity from AD dramatically increases after the age of 65 and shows a growing trend [2].

The main histological characteristic of AD is the accumulation of extracellular amyloid β (Aβ), evident as senile plaques and intracellular neurofibrillary tangles (NFTs) caused by hyperphosphorylated tau [3]. Evidence has shown that the degree of dementia is closely connected to the level of soluble oligomers of Aβ species in AD patients’ brains [4]. The Aβ oligomer, formed by redundant Aβ_42_, can contribute to the damage of ion channels and calcium homeostasis, reduced energy metabolism, and glucose regulation [4,5], as it disrupts neuronal regulation and synaptic plasticity and causes eventual neuron death.

Studies have reported that Aβ oligomers first appeared in the brain in 2-month-old APP/PS1 mice [6], senile plaques were detected in 4-month-old mice [7], and the numbers and areas of plaques increased with age. Therefore, the early treatment of AD is crucial. The classical lesions of AD can occur as early as 20 years prior to the development of symptoms and disease indicators [8]. These early changes are likely to occur at the epigenetic level, where gene expression is controlled [9]. Histone acetylation is a common form of genetic post-translational modification and plays an important role in histone transcription regulation [10].

Histone deacetylases (HDACs) are a superfamily of enzymes that are key parts of the epigenetic regulation of gene expression and cellular activity [11]. In normal neurons, histone acetyltransferase (HAT) and HDAC protein levels and their corresponding activities are always maintained at a high balance [12]. They play crucial roles in regulating gene expression, which is associated with normal neurophysiological functions such as long-term potentiation, learning, and memory [13,14]. In neurodegenerative diseases, however, acetylation homeostasis is disrupted and synaptic plasticity is injured [15,16]. The classical HDAC family can be divided into three types according to the homology of yeast: class I HDACs (HDAC1, 2, 3, and 8), class II HDACs (HDAC4, 5, 6, 7, 9, and 10), and class III HDACs [17,18]. Studies have demonstrated that HDAC2-overexpressing mice will have deregulated gene expression and damaged synaptic plasticity, learning, and memory [19], and HDAC2 KO mice have increased dendritic spine density and numbers of synapses [20]. Moreover, HDAC3 overexpression in the hippocampuses of APP/PS1 mice can increase Aβ levels, activate microglia, and injure synaptic plasticity [21,22].

HDAC inhibitors (HDACIs) represent prototypical “epigenetic” agents that act by modifying gene expression to restore the normal differentiation or death programming of transformed cells [23]. There is powerful evidence suggesting that HDACIs may be useful in the treatment of AD and AD-like pathologies [24,25]. HDACI can activate CREB-CBP-dependent transcription [11]. cAMP response element binding protein (CREB), a transcriptional coactivator with HAT activity, has been proved to be associated with synaptic plasticity and long-term memory [26,27]. Studies have suggested that HDACI improves gene transcription ability and facilitates the phosphorylation of CREB at serine 133, driving CRE-mediated transcription [13]. The CREB transcription factor family regulates the transcription of brain-derived nerve factor (BDNF), especially the activation of BDNF promoter IV [28]. Moreover, BDNF levels can affect the chronic inflammatory state of the brain by influencing the release of TNF-α and IL-1β [29]. Aβ deposition can activate microglia, which will release pro-inflammatory cytokines such as TNF-α, IL-1β, and IL-6, leading to tau hyperphosphorylation and neuronal loss [30]. Therefore, HDACI may improve synaptic plasticity by regulating inflammation. BG45, a novel class I HDAC inhibitor (C_11_H_10_N_4_O, 214.22), has been evidenced to selectively inhibit the expression of HDAC3 (IC50 = 289 nM). It also inhibits HDAC1 and HDAC2 with reduced potency [31]. 

The entorhinal cortex has been shown to be an interface between the hippocampus and neocortical regions, and it plays a crucial role in the formation and consolidation of memory [32,33]. In our previous studies, we found that BG45 can reduce Aβ deposition, increase the expression of synaptic proteins, and upregulate the expression of α-amino-3-hydroxy-5-methyl-4-isoxazole-propionic acid receptor subunits (GluR2, GluR3, and GluR4) [34,35]. We hypothesized that BG45 might be a promising factor to rescue synaptic plasticity in AD. 

In this study, we explored the timing of Aβ deposition and the increased activation of glial cells in the entorhinal cortex. Subsequently, we investigated the effect of BG45 treatment on these changes at different times. We discovered that BG45 reduced the number of degenerative neurons and the activation of microglia and astrocytes in the entorhinal cortexes of 6-month-old mice if they were treated with BG45 at 2 months of age. 

## 2. Results

### 2.1. Age-Related Changes in Aβ in the Entorhinal Cortexes of the APP/PS1 and Wt Mice

**Figure 1:**
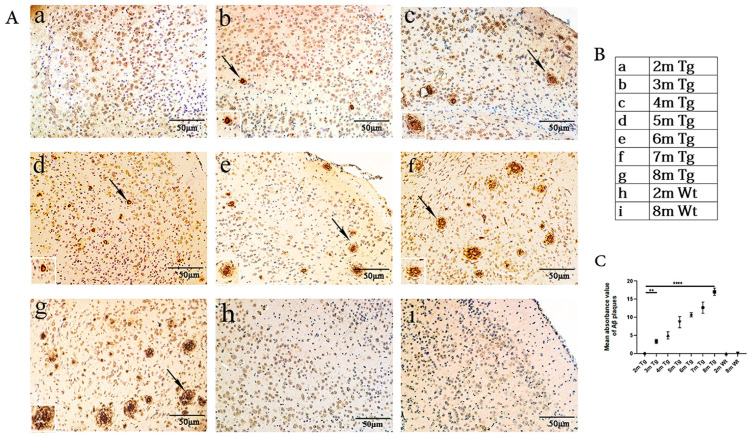
Age-related Aβ changes in the entorhinal cortexes of the APP/PS1 and Wt mice. (**Aa**–**g**) Entorhinal cortexes of APP/PS1 mice at 2–8 months (arrows show Aβ deposition). (**Ah**–**i**) Entorhinal cortexes of Wt mice at 2 and 8 months. (**B**) The details of the sample groups. (**C**) Quantitative statistical analysis of Aβ density. Scale bar = 50 μm. All values are presented as the means ± SDs from three independent experiments. **** *p* < 0.0001 and ** *p* < 0.01. *n* = five mice per group.

Immunohistochemistry revealed the differences in the Aβ depositions of the mice of different ages. Positive Aβ deposition in the entorhinal cortex was first identified in 3-month-old APP/PS1 mice, which gradually accumulated with age. However, there was no significant Aβ immunoreactivity in 2-month-old to 8-month-old Wt mice (Figure 1).

### 2.2. Age-Related Changes in Microglia and Astrocytes in the Entorhinal Cortexes of the APP/PS1 and Wt Mice

**Figure 2:**
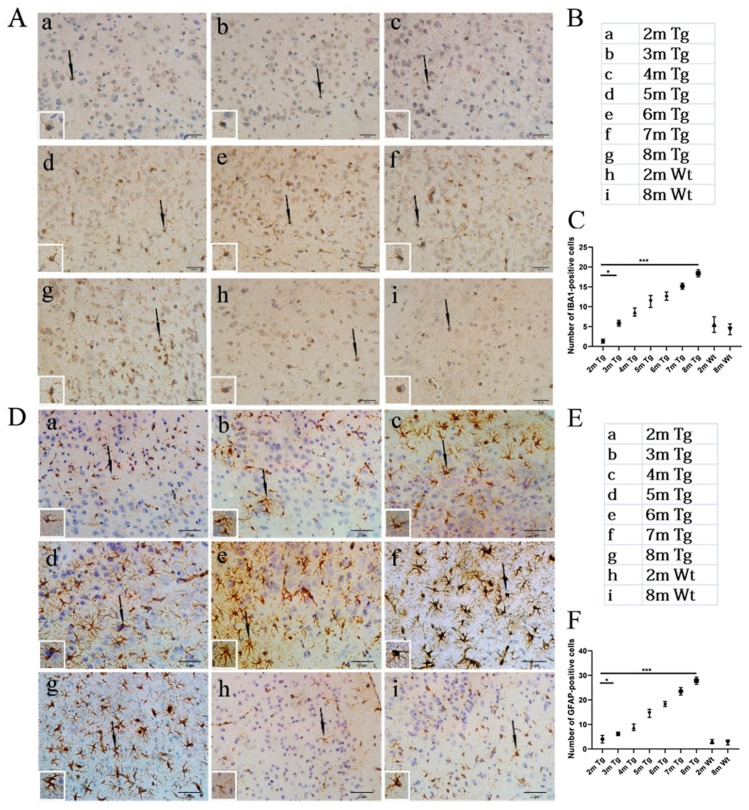
Age-related changes in the microglia and astrocytes in the entorhinal cortexes of the APP/PS1 and Wt mice. (**Aa**–**g**) Microglia in the entorhinal cortexes of the APP/PS1 mice at 2–8 months. (**Ah**,**i**) Microglia in the entorhinal cortexes of the Wt mice at 2 and 8 months. (**B**) The details of the sample groups. (**C**) Quantitative statistical analysis of microglia quality. (**Da**–**g**) Astrocytes in the entorhinal cortexes of the APP/PS1 mice at 2–8 months. (**Dh**,**i**) Astrocytes in the entorhinal cortexes of the Wt mice at 2 and 8 months. (**E**) The details of the sample groups. (**F**) Quantitative statistical analysis of astrocyte quality. Scale bar = 20 μm. All values are presented as the means ± SDs from three independent experiments. *** *p* < 0.001 and * *p* < 0.05. *n* = five mice per group.

Using immunohistochemistry, microglia and astrocytes with the corresponding IBA1 and GFAP antibodies were detected in the entorhinal cortexes at different ages. The results showed that the positive expression of IBA1 and GFAP was significantly increased in 3-month-old mice as they got older. However, compared with 2-month-old Wt mice, there were no significant changes in the IBA1-positive microglia and GFAP-positive astrocytes in the 8-month-old Wt mice (Figure 2).

### 2.3. BG45 Promoted H3K9K14/H3 Acetylation and Inhibited the Expression of HDAC1, HDAC2, and HDAC3 in the Entorhinal Cortexes of the APP/PS1 Mice 

**Figure 3:**
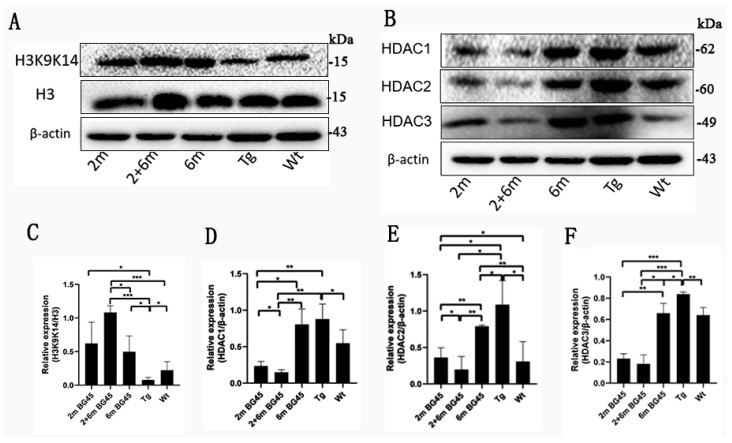
Level of H3K9K14/H3 acetylation and expression of HDAC1, HDAC2, and HDAC3 in the entorhinal cortexes of the APP/PS1 mice. (**A**) Representative blots of H3K9K14/H3 acetylation. (**B**) Representative blots of HDAC1, HDAC2, and HDAC3 expression in the entorhinal cortex samples. (**C**) Quantitative statistical analysis of H3K9K14/H3 acetylation. (**D**) Quantitative statistical analysis of HDAC1. (**E**) Quantitative statistical analysis of HDAC2. (**F**) Quantitative statistical analysis of HDAC3. All values are presented as the means ± SDs from three independent experiments. *** *p* < 0.001, ** *p* < 0.01, and * *p* < 0.05. *n* = five mice per group.

The effects of BG45, a class I HDAC inhibitor, on H3K9K14/H3 acetylation and HDAC1, HDAC2, and HDAC3 expression were evaluated by Western blot analysis. As shown in Figure 3A,C, H3K9K14/H3 acetylation was decreased in the Tg group compared with the Wt group (*p* < 0.05). However, compared with the Tg group, H3K9K14/H3 acetylation was increased to different degrees in the three BG45 treatment groups (*p* < 0.05, *p* < 0.001, and *p* < 0.05, respectively), and the levels of H3K9K14/H3 acetylation in the 2 and 6 m group were the highest. As shown in Figure 3B,D–F, HDAC1, HDAC2, and HDAC3 expression was increased in the Tg group compared with the Wt group (*p* < 0.05, *p* < 0.05, and *p* < 0.01, respectively). HDAC1 expression in the 2 m and 2 and 6 m groups was decreased compared with the Tg group (*p* < 0.01 and *p* < 0.01, respectively). However, there was no significant difference between the 6 m and Tg groups. HDAC2 and HDAC3 expression in three BG45-treated groups was decreased compared with the Tg group (*p* < 0.05, *p* < 0.05, and *p* < 0.05, respectively, and *p* < 0.001, *p* < 0.001, and *p* < 0.05, respectively).

### 2.4. BG45 Alleviated Neuronal Degeneration in the Entorhinal Cortexes of the APP/PS1 Mice

**Figure 4:**
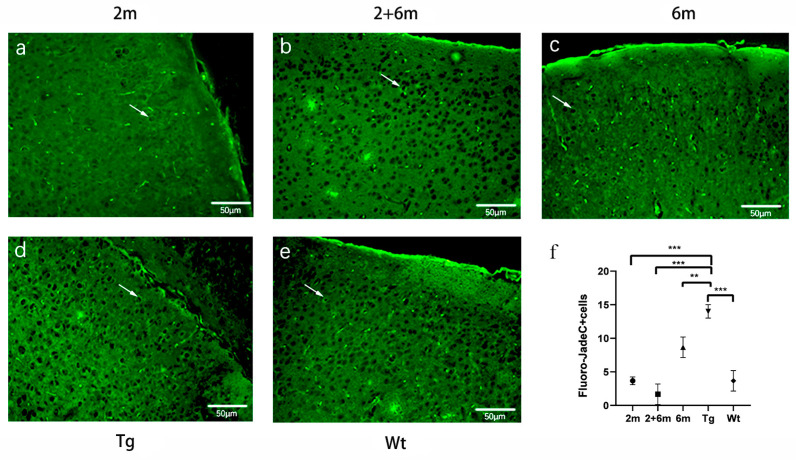
BG45 alleviated neuronal degeneration in the entorhinal cortexes of the APP/PS1 mice. (**a**–**e**) The positive FJC-stained cells were stained green (white arrows) in the entorhinal cortex region. (**f**) Quantitative statistical analysis of the FJC-positive cells. Scale bar = 50 μm. All values are presented as the means ± SDs from three independent experiments. *** *p* < 0.001 and ** *p* < 0.01. *n* = five mice per group.

The severity of neuronal degeneration was evaluated by Fluoro-Jade C (FJC) straining. We found that there were many more positive FJC-stained cells in the entorhinal cortexes of the Tg group than there were in the Wt group (*p <* 0.001). Compared with the Tg group, the number of FJC-positive cells decreased in the three groups receiving BG45 treatment (*p <* 0.001, *p <* 0.001, and *p* < 0.01) (Figure 4).

### 2.5. BG45 Reduced Aβ Deposition and Downregulated p-tau Expression in the Entorhinal Cortexes of the APP/PS1 Mice

**Figure 5:**
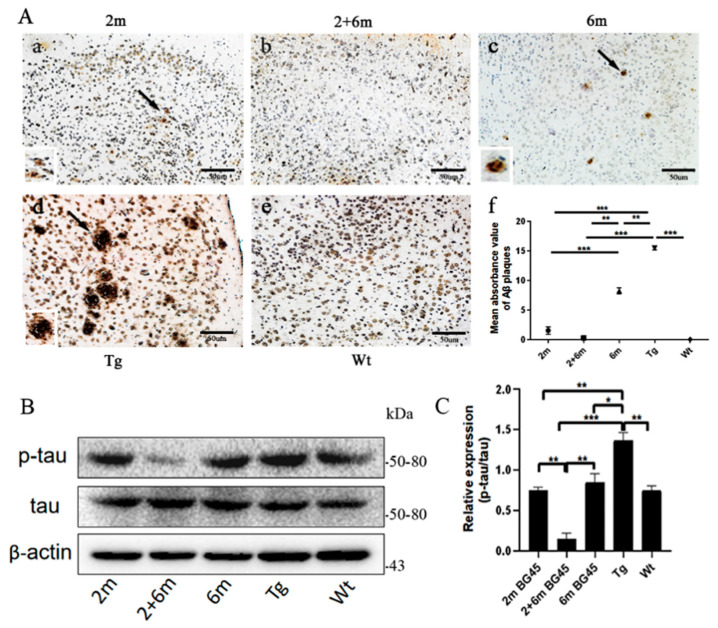
BG45 reduced Aβ deposition and downregulated p-tau expression in the entorhinal cortexes of the APP/PS1 mice. (**Aa**–**e**) Representative photographs of Aβ deposition in the entorhinal cortex samples. (**Af**) Quantitative statistical analysis of Aβ density. (**B**) Representative protein expression bands of p-tau and tau. (**C**) Quantitative statistical analysis of p-tau and tau. All values are presented as the means ± SDs from three independent experiments. *** *p* < 0.001, ** *p* < 0.01, and * *p* < 0.05. *n* = five mice per group.

As shown in Figure 5A, Aβ deposition in all BG45-treated groups (2 m, 2 and 6 m, and 6 m) was decreased compared with the Tg group (*p* < 0.001, *p* < 0.001, and *p* < 0.01, respectively). More Aβ deposition was found in the entorhinal cortexes of the mice treated at 6 months of age than in the mice treated at 2 months of age (*p* < 0.001), and the least amount was found in the mice treated twice at 2 months and 6 months of age. In addition, tau protein phosphorylation levels were also detected. The results indicated that p-tau expression was lower in all treated groups than in the Tg group (*p* < 0.01, *p* < 0.001, and *p* < 0.05, respectively), and it was lowest in the 2 and 6 m group (Figure 5B,C).

### 2.6. BG45 Increased Synaptic Protein Expression in the Entorhinal Corteesx of the APP/PS1 Mice

**Figure 6:**
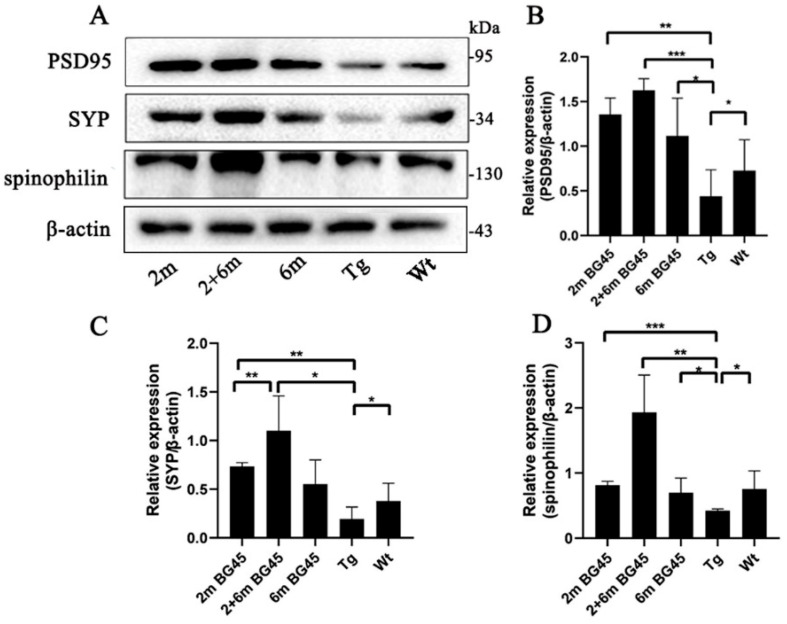
BG45 promoted the expression of synaptic proteins in the entorhinal cortexes of the APP/PS1 mice. (**A**) Representative protein expression bands of synaptic proteins. (**B**–**D**) Quantitative statistical analysis of the synaptic proteins. All values are presented as the means ± SDs from three independent experiments. *** *p* < 0.001, ** *p* < 0.01, and * *p* < 0.05. *n* = five mice per group.

To verify the protective effect of the synaptic plasticity of BG45, several synapse-related proteins were detected by Western blot analysis. The results showed that compared with the Wt group, the expression levels of PSD95, SYP, and spinophilin were decreased in the Tg group (*p* < 0.05, *p* < 0.05, and *p* < 0.05, respectively). However, the expression levels of SYP were higher in the 2 m and 2 and 6 m groups than in the Tg group (*p* < 0.01 and *p* < 0.05, respectively). In all BG45-treated groups, PSD95 and spinophilin were increased compared with the Tg group (*p* < 0.01, *p* < 0.001, and *p* < 0.05, respectively, and *p* < 0.001, *p* < 0.01, and *p* < 0.05, respectively), and PSD95 and spinophilin expression levels were higher in the 2 and 6 m group than in the two other treated groups (Figure 6).

### 2.7. BG45 Decreased the Number of IBA1-Positive Microglia and GFAP-Positive Astrocytes and Downregulated the Levels of IL-1β and TNF-α Gene Expression in the Entorhinal Cortexes of the APP/PS1 Mice

**Figure 7:**
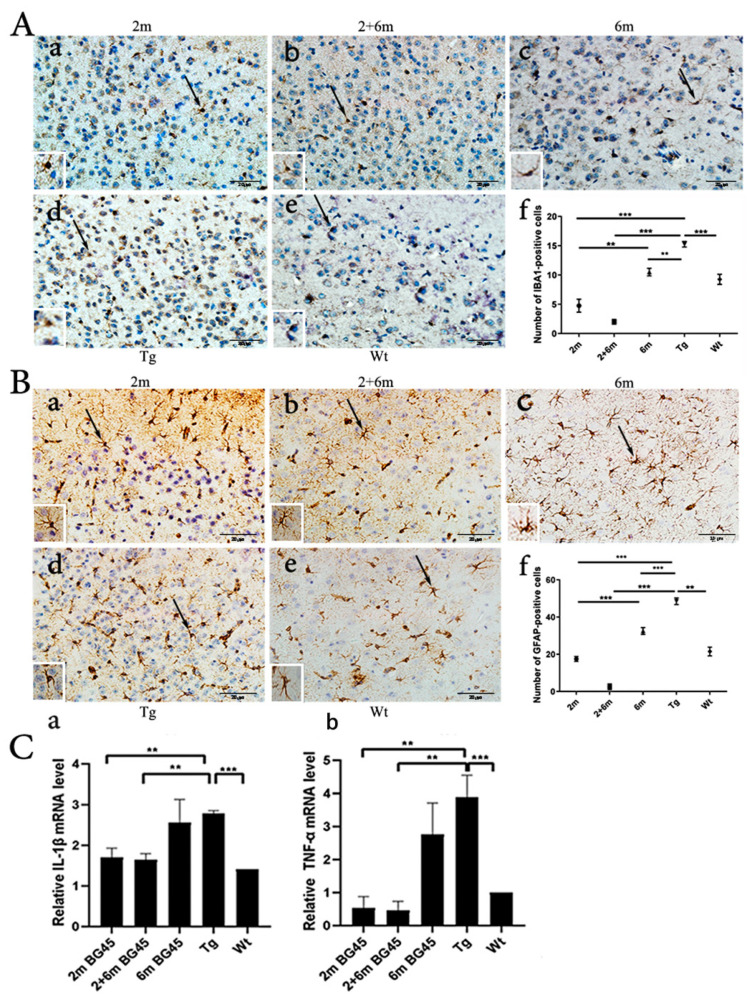
BG45 decreased the numbers of microglia and astrocytes and mRNA levels of inflammatory cytokines in the entorhinal cortexes of the APP/PS1 mice. (**Aa**–**f**) Photographs and quantitative statistical analyses of microglia in the entorhinal cortex samples. (**Ba**–**f**): Photographs and quantitative statistical analyses of astrocytes in the entorhinal cortex samples. *** *p* < 0.001 and ** *p* < 0.01. (**Ca**,**b**) mRNA levels of IL-1β and TNF-α in the entorhinal cortex samples. *** *p* < 0.001 and ** *p* < 0.01. All values are presented as the means ± SDs from three independent experiments. *n* = five mice per group.

Neuroinflammation is considered to be a critical driver of the cognitive deficits associated with AD. Overactivated neuroglial cells, such as microglia and astrocytes, contribute to neuroinflammation and neurodegenerative disorders. Both the microglia and astrocytes in the entorhinal cortexes of the 2 m, 2 and 6 m, and 6 m groups were decreased compared with the Tg group (*p* < 0.001, *p* < 0.001, and *p* < 0.01, respectively, and *p* < 0.001, *p* < 0.001, and *p* < 0.001, respectively). Moreover, the amount of IBA1-positive microglia and GFAP-positive astrocytes was the lowest in the 2 and 6 m group, and there were more positive cells in the 6 m group than in the 2 m group (*p* < 0.01 and *p* < 0.001, respectively) (Figure 7). RT-qPCR was used to detect the gene levels of inflammatory cytokines in the entorhinal cortex samples. The results showed that the mRNA levels of IL-1β and TNF-α were higher in the Tg group than in the Wt group (*p* < 0.001 and *p* < 0.001, respectively), and the levels of IL-1β and TNF-α gene expression in the 2 m and 2 and 6 m groups decreased compared with the Tg group (*p* < 0.01 and *p* < 0.01, respectively, and *p* < 0.01 and *p* < 0.01, respectively), but there was no significant difference between the 6 m group and the Tg group (Figure 7).

### 2.8. BG45 Changed the Expression of Key Factors in the CREB/BDNF/NF-kB Pathway in the Entorhinal Cortexes of the APP/PS1 Mice

**Figure 8:**
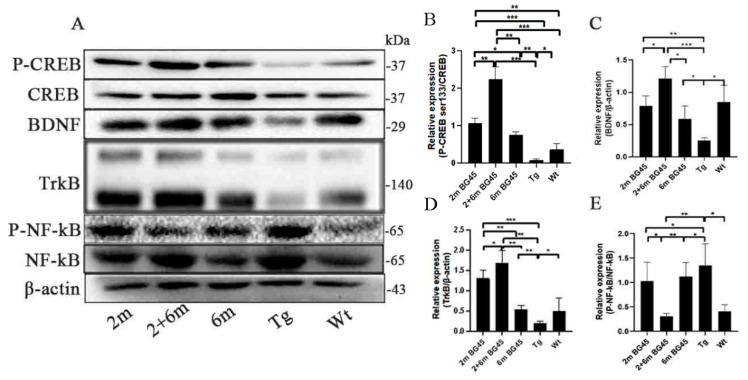
BG45 improved the expression of CREB/BDNF and inhibited p-NF-kB in the entorhinal cortexes of the APP/PS1 mice. (**A**) Representative protein expression bands of the CREB/BDNF/NF-kB pathway. (**B**–**E**) Quantitative statistical analysis of the related proteins in the entorhinal cortex samples. All values are presented as the means ± SDs from three independent experiments. *** *p* < 0.001, ** *p* < 0.01, and * *p* < 0.05. *n* = five mice per group.

In order to investigate the potential mechanism by which BG45 alleviated the inflammatory factors and improved the levels of synaptic proteins, the key factors in the CREB/BDNF/NF-kB pathway were detected. The results revealed that p-CREB/CREB, BDNF and its receptor, TrkB, and p-NF-kB/NF-kB showed significant differences between the Tg group and the Wt group. Compared with the Tg group, the levels of p-CREB/CREB in the 2 m, 2 and 6 m, and 6 m groups were upregulated (*p* < 0.001, *p* < 0.001, and *p* < 0.01, respectively), and they were the highest in the 2 and 6 m group. At the same time, BG45 upregulated the expression of BDNF and TrkB in all the treated groups compared with the Tg group (*p* < 0.01, *p* < 0.001, and *p* < 0.05, respectively, and *p* < 0.001, *p* < 0.01, and *p* < 0.01, respectively). However, the p-NF-kB/NF-kB level was inhibited by BG45 treatment. Similarly, they both showed more significant changes in the 2 and 6 m group compared with the other two treatment groups (Figure 8).

## 3. Discussion

Both amyloid precursor protein (APP) and presenilin (PSEN) gene mutations are associated with familial Alzheimer’s disease (FAD) and with the early onset of the disease. A mouse model of amyloid is used around the world to study the cognitive, behavioral, and neuropathological changes related to AD [36]. This study mainly explored the therapeutic effects of BG45 on APP/PS1 transgenic mice at different months of age before and after Aβ plaque formation.

Previous studies have found that chronic local inflammatory responses occur in pathologically vulnerable areas of the AD brain, such as the frontal lobe and hippocampus [33]. Microglia have two different effects on the development of AD. On the one hand, they can clean Aβ peptides and reduce Aβ plaque accumulation, which, in turn, protects neurons [37]. On the other hand, microglia also have a negative influence on neurons. For example, they can injure synapses and thus contribute to neuronal death by secreting inflammatory factors or activating astrocytes [37]. In this study, we found that the number of amyloid plaques consistent with IBA1-positive microglia and GFAP-positive astrocytes gradually increased from 3 to 8 months, confirming that Aβ plaques and the activation of microglia and astrocyte are closely associated in the studied region of the entorhinal cortex in AD (Figure 1 and Figure 2). 

Studies have shown that persistent epigenetic changes may affect gene expression patterns and lead to neurodegenerative disorders, including AD [38,39]. Acetylation is dysregulated in AD and associated with various impairments in signaling, inflammation, and neuronal plasticity, contributing to negatively impacted memory and cognition [40]. In a study on post-mortem AD brains, the protein levels of the total histones H3 and H4 were significantly increased [41]. Klein et al. verified the positive correlation between H3K9 acetylation and transcriptional activity in the human cortex. H3K9 acetylation level is broadly associated with tau pathology [42]. In the entorhinal cortex, AD-associated differentially acetylated peaks were enriched in some processes related to Aβ metabolic processes and synaptic proteins, and this included regions annotated to genes (APP, PSEN1, and PSEN2) involved in AD pathologic hallmarks [43]. It was reported that HDAC3 inhibitors increased histone H3 and H4 acetylation and relieved memory impairment [21]. HDAC3, a class I HDAC, plays a crucial role in the pathology of AD because it is expressed not only in the nucleus but also in the cytoplasm, unlike other HDACs. HDAC3 overexpression can impair long-term memory and lead to the death of neurons [21,22]. The previous studies proved that BG45 can rescue the expression of synaptic proteins in the prefrontal cortex of APP/PS1 transgenic mice [44]. Consistent with the situation in the hippocampus and the prefrontal cortex [34,44], in this study, our data showed that BG45 rescued synaptic proteins and neuronal degeneration in the entorhinal cortexes of APP/PS1 mice. The expression levels of HDAC1, HDAC2, and HDAC3 in the entorhinal cortexes were higher in the APP/PS1 mice than in the Wt mice (Figure 3). Further research found that BG45 effectively reduced the levels of HDAC1, HDAC2, and HDAC3 in the APP/PS1 mice by increasing the ratio of H3K9K14 to H3, i.e., the 9 and 14 double positions of histone 3, possibly resulting in dissociating histone octamers from DNA and facilitating gene transcription, as well as contributing to increased synaptic proteins, such as PSD95, spinophilin, and SYP [34]. Therefore, epigenetic mechanisms related to BG45 may contribute to gene expression events for memory and regenerative growth. 

HDACIs are a group of small molecules with HDAC-inhibitory activity and can increase the level of histone acetylation to modulate biological functions [45]. It can acetylate the lysine 9 and 14 positions of H3 of CBP, accelerating the dissociation of histone, which can promote the phosphorylation of CREB [46]. CREB, located in promoter IV of BDNF, can promote gene transcription and boost the protein level of BDNF [28,47]. Increased BDNF promotes the phosphorylation of GSK-3β at the ser9 site to inhibit GSK-3β activity through the PI3K/AKT pathway and, subsequently, tau phosphorylation at multiple sites [48,49,50]. In addition, the change in the BDNF/TrkB pathway may indicate memory deficits and injured synaptic plasticity and neurons [51,52]. At the same time, BDNF can inhibit microglia from releasing NF-kB, which can reduce the expression of TNF-α and IL-1β and the activation of microglia and astrocytes [53,54]. In this study, we detected the key factors in the CREB/BDNF/TrkB pathway in Tg mice with or without BG45 treatment. The results showed that BG45 downregulated the inflammatory cytokines TNF-α and IL-1β. The increased levels of p-CREB/CREB, BDNF, and TrkB and the reduced p-NF-kB/NF-kB levels with BG45 treatment demonstrated that the regulation of glial cells and inflammation by BG45 involves the CREB/BDNF/TrkB pathway (Figure 8). 

Recent reports have also shown that HDAC3 expression and activity are associated with the expression of several AD-related genes, pro-inflammatory TNF-α and IL-6 and GFAP [40], while the neuroprotective effect of HDAC3 inhibitor (RGFP966) on modulating neuronal memory [55] and extensive neurite outgrowth [56] increases histone H3 and H4 acetylation, reducing Aβ expression and the level of tau phosphorylation [21]. Together, these studies suggest that HDAC3 inhibitors may be a promising epigenetic therapy for AD.

Importantly, in this study, we found that some pathological changes in AD mice were better improved with BG45 treatment at the age of 2 months compared to those treated at 6 months of age, and BG45 significantly reduced the activation of microglia in the 2 and 6 m group, in which the mice were injected twice with BG45 at 2 and 6 months of age. Therefore, this demonstrated the effectiveness of early and repeated interventions with AD therapy at the epigenetic level.

In summary, the molecular mechanisms of AD are complicated. More and more studies are reporting novel hypotheses such as mitochondrial defects, the phosphorylation of tau protein, molecular genetics and etiology, inflammation, oxidative stress and free radical, virus theory, etc. However, the unbalanced production or discharge of Aβ caused by various factors, including neuroinflammation, is still the focus of AD research [57]. Amyloid β can cause neuron death by causing the leakage of ions, disruption of the cellular calcium balance, and losses in membrane potential [58]. BG45, a class I HDAC inhibitor, increased H3K9K14/H3 acetylation and alleviated the pathology of the entorhinal cortexes in APP/PS1 mice by reducing Aβ deposition and upregulating the expression of synaptic proteins. We deduced that this positive effect may be due to BG45 inhibiting the activation of microglia and astrocytes and reducing the levels of inflammatory factors through the CREB/BDNF/NF-kB pathway. 

Therefore, it is worth further studying BG45 as a promising HDAC inhibitor for the treatment of AD. To perform the omics analysis to find the interactions of the signaling pathways involved in the role of BG45 would provide more favorable evidence for its use as a therapeutic target.

## 4. Materials and Methods 

### 4.1. Animals and Drug Administration

The APP/PS1 transgenic (Tg) mice were purchased from the Nanjing Biomedical Research Institute of Nanjing University. All mice were raised under a 12 h light/dark cycle at 22 °C with free access to food and water. All procedures were approved by the Institutional Animal Care and Use Committee of Dalian Medical University (AEE18086). Wild-type C57BL/6 mice were used as normal controls (Wt group). Two-month-old male, 20–22 g APP/PS1 mice were randomly divided into 4 groups, and 3 of the groups were intraperitoneally injected with BG45 (Selleck, 926259-99-6, Houston, USA) at different times, as follows: Tg group, control; 2 m group, injected at 2 months of age; 6 m group, injected at 6 months of age; and 2 and 6 m group, separately injected at 2 and 6 months of age. The mice in all treatment groups were injected with BG45 once a day for 12 days (30 mg/kg of BG45, 0.2 mL per mouse, where the BG45 was first dissolved into a 1 mg/mL stock solution in DMSO and then diluted 1:1000 with normal saline for use). The Tg group and the Wt group were also injected with same volume of vehicle. There were 5 mice in each group. All mice were killed within 24 h after the last injection at 6 months. Their entorhinal cortexes were harvested for subsequent experiments.

### 4.2. Immunohistochemistry Staining

The sections were deparaffinized and rehydrated, and antigen retrieval was performed. Blocking endogenous peroxidase solution (SP-9100, ZSGB-BIO, Beijing, China) was added to each section, and they were incubated for 15 min at room temperature to block endogenous peroxidase [59]. The sections were blocked with a goat serum solution (SP-9100, ZSGB-BIO, Beijing, China) for 15 min at room temperature. Then the sections were incubated with primary antibody Aβ_1–42_ (NBP2-13075, Novus Biologicals, Littleton, CO, USA), IBA1 (1094-1-AP, Proteintech, Wuhan, China), or GFAP (80788, Cell Signaling Technology, Boston, MA, USA) at 4 °C overnight. After being washed with PBS (SW132-01, Seven, Beijing, China), the sections were incubated with an appropriate amount of biotin-labeled goat anti-mouse/rabbit IgG for 15 min at room temperature. Subsequently, the sections were incubated with streptozotocin-peroxidase for 15 min at room temperature. Diaminobenzidine (DAB) (ZLI-9018, ZSGB-BIO, Beijing, China) solution was added to the sections for 10 s to 5 min. Finally, hematoxylin was used to stain the nuclei [59]. Three random slices were selected from each group, and three random visual fields in the entorhinal cortex of each slice were observed [59]. The expression of Aβ_1–42_, IBA1, and GFAP was quantified by ImageJ software (U. S. National Institutes of Health, Bethesda, MD, USA).

### 4.3. Western Blotting

All samples from the entorhinal cortex were thawed and washed in PBS buffer (SW132-01, Seven, Beijing, China). Then, the samples were sonicated in RIPA lysis buffer (SW104, Seven, Beijing, China) and incubated on ice for 30 min [60]. The proteins were extracted by centrifugation at 10,000× *g* for 10 min at 4 °C, and the concentrations were detected using a BCA Protein Assay Kit (P0010, Beyotime Biotechnology, Shanghai, China). The proteins were separated by 10 or 12% SDS PAGE and transferred to polyvinylidene difluoride membranes, which were blocked in 5% skim milk. Next, the membranes were incubated with rabbit polyclonal antibodies synaptophysin (SYP) (ab32127, Abcam, London, UK); BDNF (ab108919, Abcam, London, UK); P-CREB/CREB (9197, Cell Signaling Technology, Boston, MA, USA; ab32096, Abcam, London, UK); postsynaptic density protein 95 (PSD-95) (ab18258, Abcam, London, UK); p-tau (Ser404) and tau (20194 and 46687, Cell Signaling Technology, Boston, MA, USA); spinophilin (ab18561, Abcam); HDAC1, HDAC2, and HDAC3 (34589, 57156, and 85057, Cell Signaling Technology, Boston, MA, USA); H3K9K14 (GTX122648, GeneTex, San Antonio, TX, USA); H3 (17168-1-AP, Proteintech, Wuhan, China); NF-kB and p-NF-kB (Ser536) (10745-1-AP, Proteintech, Wuhan, China; 3033, Cell Signaling Technology, Boston, MA, USA); TrkB (4603, Cell Signaling Technology, Boston, MA, USA); and β-actin (AC026, ABclone, Wuhan, China) for 12 h at 4 °C. Then, the membranes were blotted with horseradish peroxidase (HRP)-conjugated secondary antibody at the room temperature for 1 h and imaged using a ChemiDoc XRS System and Image Lab software (Bio-Rad Laboratories, Inc., Hercules, CA, USA).

### 4.4. Fluoro-Jade C Staining

The sections were dewaxed per the immunohistochemistry protocol and washed with ddH_2_O twice for 1 min each time [61]. The dilution, which was mixed with solution B (potassium permanganate, Merck Millipore, Massachusetts, USA) and ddH_2_O (1:9), was added to each section, and the sections were incubated in the dark for 10 min. Then, the solution was replaced with 0.5% Triton for 30 min. The sections were washed with ddH_2_O twice for 1 min each time. Then, one part of solution C (Fluoro-Jade C) was mixed with 1 part of DAPI and 8 parts of ddH_2_O, and the mixture was added to the slices to incubate for 10 min in the dark. After being washed 3 times for 1 min each time, the slices were placed in a drying oven at 50–60 °C for 5 min. The dried sections were immersed in xylene for at least 5 min. The sections were observed by a Nikon Eclipse 800 microscope. Three random slices were selected from each group, and for each slice, 3 random fields were counted. The data are expressed as the numbers of degenerative neurons.

### 4.5. Real-Time Quantitative Polymerase Chain Reaction

All samples were homogenated by TRIzol reagent (Takara, Kyoto, Japan). After adding chloroform to accelerate the RNA extraction, the solution was centrifuged for separation. The supernatant was transferred into an EP tube and isopropanol was added to make the RNA precipitate. After washing precipitate three times with the 75% ethyl alcohol, the precipitate was dried at room temperature. The concentration of RNA was measured, then transcribed using a reverse transcription kit (Transgene, Strasbourg, France). Samples of mRNA were added to the solution, which was mixed with primer and 2× TransStart Top Green qPCR SuperMix (Transgene, Strasbourg, France), amounting to 20 µL. The qPCR reaction system was operated according to the manufacture’s protocol (Transgene, AQ601, Strasbourg, France) (30 s at 94 °C and 5 s at 95 °C, followed by 45 cycles for 30 s at 60 °C).

The primer sequences were as follows:

TNF-α 5-GACGTGGAACTGGCAGAAGAG-3
          5-TTGGTGGTTTGTGAGTGTGAG-3
IL-1β 5-GCCCATCCTCTGTGACTCAT-3
       5-AGGCCACAGGTATTTTGTCG-3
GAPDH 5-GAGCCCTTCCACAATGCCAAAGTT-3
             5-TGTGATGGGTGTGAACCACGAGAA-3

### 4.6. Statistical Analysis

All values are expressed as means ± standard deviations (SDs) from three independent experiments. One-way analysis of variance (ANOVA) followed by Tukey’s post hoc tests were used to analyze the differences between means of several subgroups of a variable, and a *t*-test was used for comparisons between two groups using GraphPad Prism8 (GraphPad Software, La Jolla, CA, USA). Significance was accepted at *p* < 0.05.

## Data Availability

All data supporting this study are available in the article.

## References

[1] Barnes J., Dickerson B.C., Frost C., Jiskoot L.C., Wolk D., van der Flier W.M. (2015). Alzheimer’s disease first symptoms are age dependent: Evidence from the NACC dataset. Alzheimers Dement..

[2] (2020). Alzheimer’s association in USA 2020, Alzheimer’s disease facts and figures. Alzheimers Dement..

[3] Oboudiyat C., Glazer H., Seifan A., Greer C., Isaacson R.S. (2013). Alzheimer’s Disease. Semin. Neurol..

[4] Janelsins M.C., Mastrangelo M.A., Oddo S., LaFerla F.M., Federoff H.J., Bowers W.J. (2005). Early correlation of microglial activation with enhanced tumor necrosis factor-alpha and monocyte chemoattractant protein-1 expression specifically within the entorhinal cortex of triple transgenic Alzheimer’s disease mice. J. Neuroinflamm.

[5] Belfiore R., Rodin A., Ferreira E., Velazquez R., Branca C., Caccamo A., Oddo S. (2019). Temporal and regional progression of Alzheimer’s disease-like pathology in 3xTg-AD mice. Aging Cell.

[6] Duffy A.M., Morales-Corraliza J., Bermudez-Hernandez K.M., Schaner M.J., Magagna-Poveda A., Mathews P.M., Scharfman H.E. (2015). Entorhinal cortical defects in Tg2576 mice are present as early as 2–4 months of age. Neurobiol. Aging.

[7] Garcia-Alloza M., Robbins E.M., Zhang-Nunes S.X., Purcell S.M., Betensky R.A., Raju S., Prada C., Greenberg S.M., Bacskai B.J., Frosch M.P. (2006). Characterization of amyloid deposition in the APPswe/PS1dE9 mouse model of Alzheimer disease. Neurobiol. Dis..

[8] Alzheimers A. (2012). 2012 Alzheimer’s disease facts and figures. Alzheimers Dement..

[9] Nikolac Perkovic M., Videtic Paska A., Konjevod M., Kouter K., Svob Strac D., Nedic Erjavec G., Pivac N. (2021). Epigenetics of Alzheimer’s Disease. Biomolecules.

[10] Fukuda H., Sano N., Muto S., Horikoshi M. (2006). Simple histone acetylation plays a complex role in the regulation of gene expression. Brief. Funct. Genom. Proteom..

[11] Wu X., Chen P.S., Dallas S., Wilson B., Block M.L., Wang C.C., Kinyamu H., Lu N., Gao X., Leng Y. (2008). Histone deacetylase inhibitors up-regulate astrocyte GDNF and BDNF gene transcription and protect dopaminergic neurons. Int. J. Neuropsychopharmacol..

[12] Peserico A., Simone C. (2011). Physical and Functional HAT/HDAC Interplay Regulates Protein Acetylation Balance. J. Biomed. Biotechnol..

[13] Wey H.Y., Gilbert T.M., Zürcher N.R., She A., Bhanot A., Taillon B.D., Schroeder F.A., Wang C., Haggarty S.J., Hooker J.M. (2016). Insights into neuroepigenetics through human histone deacetylase PET imaging. Sci. Transl. Med..

[14] Anderson K.W., Chen J., Wang M., Mast N., Pikuleva I.A., Turko I.V. (2015). Quantification of Histone Deacetylase Isoforms in Human Frontal Cortex, Human Retina, and Mouse Brain. PLoS ONE.

[15] Sharma S., Sarathlal K.C., Taliyan R. (2019). Epigenetics in Neurodegenerative Diseases: The Role of Histone Deacetylases. CNS Neurol. Disord. Drug Targets.

[16] Gupt R., Ambast R.K., Kumar P. (2021). Histone deacetylase in neuropathology. Adv. Clin. Chem..

[17] de Ruijter A.J., van Gennip A.H., Caron H.N., Kemp S., van Kuilenburg A.B. (2003). Histone deacetylases (HDACs): Characterization of the classical HDAC family. Biochem. J..

[18] Kumar V., Kundu S., Singh A., Singh S. (2022). Understanding the Role of Histone Deacetylase and their Inhibitors in Neurodegenerative Disorders: Current Targets and Future Perspective. Curr. Neuropharmacol..

[19] Yamakawa H., Cheng J., Penney J., Gao F., Rueda R., Wang J., Yamakawa S., Kritskiy O., Gjoneska E., Tsai L.H. (2017). The Transcription Factor Sp3 Cooperates with HDAC2 to Regulate Synaptic Function and Plasticity in Neurons. Cell Rep..

[20] Guan J.S., Haggarty S.J., Giacometti E., Dannenberg J.H., Joseph N., Gao J., Nieland T.J., Zhou Y., Wang X., Mazitschek R. (2009). HDAC2 negatively regulates memory formation and synaptic plasticity. Nature.

[21] Janczura K.J., Volmar C.H., Sartor G.C., Rao S.J., Ricciardi N.R., Lambert G., Brothers S.P., Wahlestedt C. (2018). Inhibition of HDAC3 reverses Alzheimer’s disease-related pathologies in vitro and in the 3xTg-AD mouse model. Proc. Natl. Acad. Sci. USA.

[22] Zhu X., Wang S., Yu L., Jin J., Ye X., Liu Y., Xu Y. (2017). HDAC3 negatively regulates spatial memory in a mouse model of Alzheimer’s disease. Aging Cell.

[23] Bose P., Dai Y., Grant S. (2014). Histone deacetylase inhibitor (HDACI) mechanisms of action: Emerging insights. Pharmacol. Ther..

[24] Gräff J., Rei D., Guan J.S., Wang W.Y., Seo J., Hennig K.M., Nieland T.J., Fass D.M., Kao P.F., Kahn M. (2012). An epigenetic blockade of cognitive functions in the neurodegenerating brain. Nature.

[25] Takada N., Nakamura Y., Ikeda K., Takaoka N., Hisaoka-Nakashima K., Sanoh S., Kotake Y., Nakata Y., Morioka N. (2021). Treatment with Histone Deacetylase Inhibitor Attenuates Peripheral Inflammation-Induced Cognitive Dysfunction and Microglial Activation: The Effect of SAHA as a Peripheral HDAC Inhibitor. Neurochem. Res..

[26] Kaldun J.C., Sprecher S.G. (2019). Initiated by CREB: Resolving Gene Regulatory Programs in Learning and Memory Switch in Cofactors and Transcription Regulators between Memory Consolidation and Maintenance Network. Bioessays.

[27] Benito E., Barco A. (2010). CREB’s control of intrinsic and synaptic plasticity: Implications for CREB-dependent memory models. Trends Neurosci..

[28] Esvald E.E., Tuvikene J., Sirp A., Patil S., Bramham C.R., Timmusk T. (2020). CREB Family Transcription Factors Are Major Mediators of BDNF Transcriptional Autoregulation in Cortical Neurons. J. Neurosci..

[29] Bi Q.R., Hou J.J., Qi P., Ma C.H., Shen Y., Feng R.H., Yan B.P., Wang J.W., Shi X.J., Zheng Y.Y. (2016). Venenum Bufonis induces rat neuroinflammation by activiating NF-κB pathway and attenuation of BDNF. J. Ethnopharmacol..

[30] Lima Giacobbo B., Doorduin J., Klein H.C., Dierckx R.A.J.O., Bromberg E., de Vries E.F.J. (2019). Brain-Derived Neurotrophic Factor in Brain Disorders: Focus on Neuroinflammation. Mol. Neurobiol..

[31] Minami J., Suzuki R., Mazitschek R., Gorgun G., Ghosh B., Cirstea D., Hu Y., Mimura N., Ohguchi H., Cottini F. (2014). Histone deacetylase 3 as a novel therapeutic target in multiple myeloma. Leukemia.

[32] Takehara-Nishiuchi K. (2014). Entorhinal cortex and consolidated memory. Neurosci. Res..

[33] Pennanen C., Kivipelto M., Tuomainen S., Hartikainen P., Hänninen T., Laakso M.P., Hallikainen M., Vanhanen M., Nissinen A., Helkala E.L. (2004). Hippocampus and entorhinal cortex in mild cognitive impairment and early AD. Neurobiol. Aging.

[34] Han Y., Chen L., Liu J., Chen J., Wang C., Guo Y., Yu X., Zhang C., Chu H., Ma H. (2022). A Class I HDAC Inhibitor Rescues Synaptic Damage and Neuron Loss in APP-Transfected Cells and APP/PS1 Mice through the GRIP1/AMPA Pathway. Molecules.

[35] Han Y., Chen L., Guo Y., Wang C., Zhang C., Kong L., Ma H. (2021). Class I HDAC Inhibitor Improves Synaptic Proteins and Repairs Cytoskeleton Through Regulating Synapse-Related Genes In vitro and In vivo. Front. Aging Neurosci..

[36] Kosel F., Pelley J.M.S., Franklin T.B. (2020). Behavioural and psychological symptoms of dementia in mouse models of Alzheimer’s disease-related pathology. Neurosci. Biobehav. Rev..

[37] Hansen D.V., Hanson J.E., Sheng M. (2018). Microglia in Alzheimer’s disease. J. Cell Biol..

[38] Delgado-Morales R., Esteller M. (2017). Opening up the DNA methylome of dementia. Mol. Psychiatry.

[39] Lahiri D.K., Maloney B., Zawia N.H. (2009). The LEARn model: An epigenetic explanation for idiopathic neurobiological diseases. Mol. Psychiatry.

[40] Martínez-Iglesias O., Naidoo V., Carrera I., Cacabelos R. (2022). Epigenetic Studies in the Male APP/BIN1/COPS5 Triple-Transgenic Mouse Model of Alzheimer’s Disease. Int. J. Mol. Sci..

[41] Narayan P.J., Lill C., Faull R., Curtis M.A., Dragunow M. (2015). Increased acetyl and total histone levels in post-mortem Alzheimer’s disease brain. Neurobiol. Dis..

[42] Klein H.U., McCabe C., Gjoneska E., Sullivan S.E., Kaskow B.J., Tang A., Smith R.V., Xu J., Pfenning A.R., Bernstein B.E. (2019). Epigenome-wide study uncovers large-scale changes in histone acetylation driven by tau pathology in aging and Alzheimer’s human brains. Nat. Neurosci..

[43] Marzi S.J., Leung S.K., Ribarska T., Hannon E., Smith A.R., Pishva E., Poschmann J., Moore K., Troakes C., Al-Sarraj S. (2018). A histone acetylome-wide association study of Alzheimer’s disease identifies disease-associated H3K27ac differences in the entorhinal cortex. Nat. Neurosci..

[44] Liu J., Zhang C., Wang J., Huang Y., Shen D., Hu Y., Chu H., Yu X., Zhang L., Ma H. (2022). A Class I HDAC Inhibitor BG45 Alleviates Cognitive Impairment through the CaMKII/ITPKA/Ca Signaling Pathway. Pharmaceuticals.

[45] Wang J., Yun F., Sui J., Liang W., Shen D., Zhang Q. (2022). HAT- and HDAC-Targeted Protein Acetylation in the Occurrence and Treatment of Epilepsy. Biomedicines.

[46] Citraro R., Leo A., De Caro C., Nesci V., Gallo Cantafio M.E., Amodio N., Mattace Raso G., Lama A., Russo R., Calignano A. (2020). Effects of Histone Deacetylase Inhibitors on the Development of Epilepsy and Psychiatric Comorbidity in WAG/Rij Rats. Mol. Neurobiol..

[47] Amidfar M., de Oliveira J., Kucharska E., Budni J., Kim Y.K. (2020). The role of CREB and BDNF in neurobiology and treatment of Alzheimer’s disease. Life Sci..

[48] Zhang H., Han Y., Zhang L., Jia X., Niu Q. (2021). The GSK-3β/β-Catenin Signaling-Mediated Brain-Derived Neurotrophic Factor Pathway Is Involved in Aluminum-Induced Impairment of Hippocampal LTP In Vivo. Biol. Trace Elem. Res..

[49] Yang W., Liu Y., Xu Q.Q., Xian Y.F., Lin Z.X. (2020). Sulforaphene Ameliorates Neuroinflammation and Hyperphosphorylated Tau Protein via Regulating the PI3K/Akt/GSK-3 beta Pathway in Experimental Models of Alzheimer’s Disease. Oxidative Med. Cell. Longev..

[50] Elliott E., Atlas R., Lange A., Ginzburg I. (2005). Brain-derived neurotrophic factor induces a rapid dephosphorylation of tau protein through a PI-3Kinase signalling mechanism. Eur. J. Neurosci..

[51] Yoshii A., Constantine-Paton M. (2010). Postsynaptic BDNF-TrkB Signaling in Synapse Maturation, Plasticity, and Disease. Dev. Neurobiol..

[52] Andero R., Choi D., Ressler K. (2014). BDNF-TrkB receptor regulation of distributed adult neural plasticity, memory formation, and psychiatric disorders. Prog. Mol. Biol. Transl. Sci..

[53] Chen J.J., Wang T., An C.D., Jiang C.Y., Zhao J., Li S. (2016). Brain-derived neurotrophic factor: A mediator of inflammation-associated neurogenesis in Alzheimer’s disease. Rev. Neurosci..

[54] Luo H., Xiang Y., Qu X., Liu H., Liu C., Li G., Han L., Qin X. (2019). Apelin-13 Suppresses Neuroinflammation Against Cognitive Deficit in a Streptozotocin-Induced Rat Model of Alzheimer’s Disease Through Activation of BDNF-TrkB Signaling Pathway. Front. Pharmacol..

[55] Phan M.L., Gergues M.M., Mahidadia S., Jimenez-Castillo J., Vicario D.S., Bieszczad K.M. (2017). HDAC3 Inhibitor RGFP966 Modulates Neuronal Memory for Vocal Communication Signals in a Songbird Model. Front. Syst. Neurosci..

[56] Hervera A., Zhou L., Palmisano I., McLachlan E., Kong G., Hutson T.H., Danzi M.C., Lemmon V.P., Bixby J.L., Matamoros-Angles A. (2019). PP4-dependent HDAC3 dephosphorylation discriminates between axonal regeneration and regenerative failure. EMBO J..

[57] Soria Lopez J.A., Gonzalez H.M., Leger G.C. (2019). Alzheimer’s disease. Handb. Clin. Neurol..

[58] Reiss A.B., Arain H.A., Stecker M.M., Siegart N.M., Kasselman L.J. (2018). Amyloid toxicity in Alzheimer’s disease. Rev. Neurosci..

[59] Guo Y., Zhang C., Wang C., Huang Y., Liu J., Chu H., Ren X., Kong L., Ma H. (2021). Thioredoxin-1 Is a Target to Attenuate Alzheimer-Like Pathology in Diabetic Encephalopathy by Alleviating Endoplasmic Reticulum Stress and Oxidative Stress. Front. Physiol..

[60] Huang Q., Zhang C., Dong S., Han J., Qu S., Xie T., Zhao H., Shi Y. (2022). Asafoetida exerts neuroprotective effect on oxidative stress induced apoptosis through PI3K/Akt/GSK3 beta/Nrf2/HO-1 pathway. Chin. Med..

[61] Ikenari T., Kurata H., Satoh T., Hata Y., Mori T. (2020). Evaluation of Fluoro-Jade C Staining: Specificity and Application to Damaged Immature Neuronal Cells in the Normal and Injured Mouse Brain. Neuroscience.

